# Association of the gut microbiome with kidney function and damage in the Hispanic Community Health Study/Study of Latinos (HCHS/SOL)

**DOI:** 10.1080/19490976.2023.2186685

**Published:** 2023-03-07

**Authors:** Brandilyn A. Peters, Qibin Qi, Mykhaylo Usyk, Martha L. Daviglus, Jianwen Cai, Nora Franceschini, James P. Lash, Marc D. Gellman, Bing Yu, Eric Boerwinkle, Rob Knight, Robert D. Burk, Robert C. Kaplan

**Affiliations:** a Department of Epidemiology and Population Health, Albert Einstein College of Medicine, Bronx, NY, USA; b Department of Microbiology and Immunology, Albert Einstein College of Medicine, Bronx, NY, USA; c Institute for Minority Health Research, University of Illinois at Chicago, Chicago, IL, USA; d Department of Biostatistics, Gillings School of Global Public Health, University of North Carolina, Chapel Hill, NC, USA; e Department of Epidemiology, Gillings School of Global Public Health, University of North Carolina, Chapel Hill, NC, USA; f Department of Medicine, University of Illinois, Chicago, IL, USA; g Department of Psychology, University of Miami, Miami, FL, USA; h Department of Epidemiology, Human Genetics, and Environmental Sciences, School of Public Health, The University of Texas Health Science Center at Houston, Houston, TX, USA; i Departments of Pediatrics, Computer Science and Engineering, Bioengineering, and Center for Microbiome Innovation, University of California San Diego, La Jolla, CA, USA; j Department of Obstetrics & Gynecology and Women’s Health, Albert Einstein College of Medicine, Bronx, NY, USA; k Public Health Sciences Division, Fred Hutchinson Cancer Research Center, Seattle, WA, USA

**Keywords:** Gut microbiome, chronic kidney disease, glomerular filtration rate, metabolites

## Abstract

**Background:**

The gut microbiome is altered in chronic kidney disease (CKD), potentially contributing to CKD progression and co-morbidities, but population-based studies of the gut microbiome across a wide range of kidney function and damage are lacking.

**Methods:**

In the Hispanic Community Health Study/Study of Latinos, gut microbiome was assessed by shotgun sequencing of stool (*n* = 2,438; 292 with suspected CKD). We examined cross-sectional associations of estimated glomerular filtration rate (eGFR), urinary albumin:creatinine (UAC) ratio, and CKD with gut microbiome features. Kidney trait-related microbiome features were interrogated for correlation with serum metabolites (*n* = 700), and associations of microbiome-related serum metabolites with kidney trait progression were examined in a prospective analysis (*n* = 3,635).

**Results:**

Higher eGFR was associated with overall gut microbiome composition, greater abundance of species from Prevotella, Faecalibacterium, Roseburia, and Eubacterium, and microbial functions related to synthesis of long-chain fatty acids and carbamoyl-phosphate. Higher UAC ratio and CKD were related to lower gut microbiome diversity and altered overall microbiome composition only in participants without diabetes. Microbiome features related to better kidney health were associated with many serum metabolites (e.g., higher indolepropionate, beta-cryptoxanthin; lower imidazole propionate, deoxycholic acids, p-cresol glucuronide). Imidazole propionate, deoxycholic acid metabolites, and p-cresol glucuronide were associated with prospective reductions in eGFR and/or increases in UAC ratio over ~6 y.

**Conclusions:**

Kidney function is a significant correlate of the gut microbiome, while the relationship of kidney damage with the gut microbiome depends on diabetes status. Gut microbiome metabolites may contribute to CKD progression.

## Introduction

Chronic kidney disease (CKD) afflicts approximately 15% of the U.S. population^1^. CKD, defined as either kidney damage, generally indicated by urinary albumin, or decreased kidney function, measured by glomerular filtration rate (GFR), is associated with increased mortality^2^ and cardiovascular disease (CVD)^3^, and reduced health-related quality of life^4^. Prevention and management of CKD risk factors are vital to reduce CKD-related morbidity and mortality.

Known causes of CKD include hypertension, autoimmune diseases, diabetes, infections, or drug toxicity, while susceptibility to CKD is influenced by age, obesity, and genetics^5^. The gut microbiota, the community of microorganisms residing in the human gut, may also be important modulators of CKD risk. Many studies have shown an altered gut microbiome in patients with CKD compared to healthy controls^6^, perhaps attributed to CKD itself, though microbial metabolic activities, particularly the synthesis of uremic toxins, may contribute to CKD progression and CVD^7–11^.

Bi-directional relationships of kidney function and the gut microbiome are termed the “gut-kidney axis.” In health, gut microbiota maintain intestinal barrier integrity, preventing translocation of pathogens and inflammatory microbial products to the circulation^12^, and ferment polysaccharides into beneficial short-chain fatty acids (SCFAs), which may protect from CKD progression^13^. In CKD, there is increased translocation and retention of gut microbiome-derived products of protein fermentation, such as p-cresol sulfate, indoxyl sulfate, and phenylacetylglutamine, and these uremic toxins confer renal and cardiovascular toxicity^9,12^. Regarding the impact of CKD on the gut microbiota, increased gut urea secretion in CKD can lead to gut barrier degradation and overgrowth of urease-containing bacteria^8,9^, and patients with CKD may alter their diet and use medications which can also alter their microbiota. Gut microbiome dysbiosis caused by CKD is thought to further degrade the intestinal barrier, reduce SCFA production, and favor uremic toxin production^9,12,14^, implying a cyclical interaction wherein dysbiosis enhances CKD progression, increased CKD severity promotes further dysbiosis, and so on.

**Figure 1.**
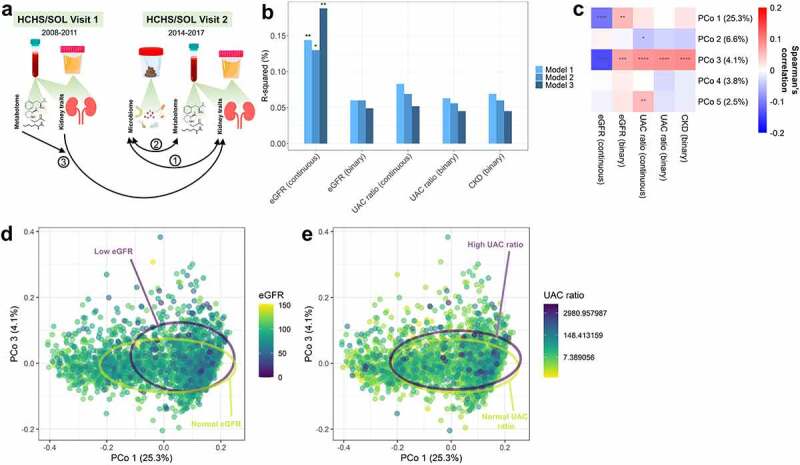
Estimated glomerular filtration rate (eGFR) is associated with overall gut microbiome composition in the Hispanic Community Health Study/Study of Latinos (*N* = 2,438). (a) Overview of the present analyses: 1 – Cross-sectional analysis of the gut microbiome and kidney traits at HCHS/SOL Visit 2 (*n* = 2,438); 2 – Cross-sectional analysis of kidney-related gut microbiome features with serum metabolites at HCHS/SOL Visit 2 (*n* = 700); 3 – Prospective analysis of serum metabolites (specifically those strongly associated with kidney-related gut microbiome species) at HCHS/SOL Visit 1 with progression of kidney traits from Visit 1 to Visit 2 (*n* = 3,635). (b) Barplots show R-squared (%) for kidney traits from PERMANOVA models of the Jensen-Shannon Divergence. Each kidney trait (predictor) was assessed in a separate model. Model 1 was adjusted for age, sex, field center, Hispanic/Latino background, U.S. nativity, antibiotics use, and Bristol stool type. Model 2 additionally adjusted for income, educational attainment, cigarette smoking, alcohol use, AHEI2010, predicted sodium intake, report of low-sodium diet, predicted protein intake, report of high protein/low carb diet, protein supplement use, and total physical activity. Model 3 additionally adjusted for BMI, waist-to-hip ratio, systolic blood pressure, diastolic blood pressure, triglycerides, HDL cholesterol, fasting glucose, hypertension medication, diabetes medication, and lipid-lowering medication. (c) Spearman correlations of kidney traits with the first five principal coordinates of the Jensen-Shannon Divergence. (d-e) Principal coordinate analysis plots of the first and third coordinates of the Jensen-Shannon Divergence, colored by eGFR in (d) or UAC ratio in (e); 75% data ellipses for low (<60 ml/min/1.73 m^2^) and normal (≥60 ml/min/1.73 m^2^) eGFR in (d), or high (≥30) or normal (<30) UAC ratio in (e), are displayed on the plots. *p < 0.05; **p < 0.01; ***p < 0.001; ****p < 0.0001.

Previous investigations on the gut microbiome and CKD have been small (*N* < 300) case–control studies, many focused on end-stage renal disease (ESRD) rather than the entire range of kidney function and damage^6^. We sought to identify patterns of gut microbiome composition related to kidney function and damage, by assessing cross-sectional relationships of gut microbiome species and functions with estimated GFR (eGFR), urinary albumin:creatinine (UAC) ratio, and CKD in the large Hispanic Community Health Study/Study of Latinos (HCHS/SOL). High prevalence of diabetes in the study population allowed us to examine possible effect modification by diabetes status, important given known pathological differences in diabetic and non-diabetic kidney disease^15^. We additionally explored cross-sectional associations of kidney-related microbiome features with serum metabolites and investigated prospective associations of specific microbiome-related metabolites with change in eGFR and UAC ratio over time and incidence of CKD (Figure 1a).

## Results

### Participant characteristics

Among 2,438 participants (Supplementary Figure S1), 292 (12%) were classified as having CKD (134, 76, 73, 7, and 2 with stages 1–5 CKD, respectively). Participants with CKD were significantly older than participants without CKD, had higher levels of CKD risk factors including blood pressure and fasting glucose, and were more likely to be taking medication for hypertension, diabetes, and high cholesterol (all *p* < 0.05; Table 1). We also observed these differences comparing participants with low and normal eGFR (Supplementary Table S1), and with and without albuminuria (Supplementary Table S2). Participants with diabetes (*n* = 698) had significantly lower eGFR, higher UAC ratio, and higher prevalence of CKD than participants without diabetes (*n* = 1740) (Supplementary Table S3).

**Table 1.** Characteristics of participants with and without chronic kidney disease (CKD)^a^ in the HCHS/SOL Gut Origins of Latino Diabetes ancillary study.

	CKD	No CKD	P-value^b^
**N**	292	2146	
**Age, years (mean ± SD)**	60.2 ± 10.5	55.6 ± 10.9	<0.0001
**Male (%)**	39.7	33.2	0.03
**Field center (%)**			0.37
Bronx	29.1	24.7	
Chicago	28.1	28.2	
Miami	17.8	20.6	
San Diego	25	26.4	
**Hispanic/Latino background (%)**			0.0002
Dominican	6.2	10.5	
Central American	7.2	9.4	
Cuban	12.3	13.4	
Mexican	41.8	42.4	
Puerto Rican	26.7	16	
South American	5.1	7.1	
Mixed/missing	0.7	1.3	
**U.S. nativity (%)**	13.7	14	0.95
**Antibiotics in last 6 months (%)**	28.1	27.4	0.87
**Income (%)**			0.006
Less than $30000	63.4	56.2	
$30000 or more	30.1	39.3	
Missing	6.5	4.5	
**Educational attainment (%)**			0.0002
Less than HS	46.9	34.4	
HS or equivalent	20.2	21.8	
More than HS	31.5	42.7	
Missing	1.4	1.1	
**Cigarette smoking (%)**			0.08
Never	57.2	63.7	
Former	26.4	23.1	
Current	16.4	13.2	
**Alcohol use (%)**			0.04
Nondrinker	52.7	44.8	
Low-level use	44.5	51.8	
High-level use	2.7	3.4	
**Alternate Healthy Eating Index 2010 score (mean ± SD)**	50.7 ± 7.9	50.3 ± 7.5	0.63
**Predicted sodium intake, mg/d (mean ± SD)**	2928 ± 850.7	2985.3 ± 843.9	0.26
**Low sodium diet (%)**	27.4	23.5	0.16
**Predicted protein intake, g/d (mean ± SD)**	76.0 ± 18.9	76.0 ± 18.2	0.76
**High protein/low carb diet (%)**	17.1	15.0	0.38
**Protein supplements (%)**	0.3	2.8	0.02
**GPAQ total physical activity, MET-min/d (mean ± SD)**	461.9 ± 817.3	573.1 ± 908.8	0.02
**BMI, kg/m2 (mean ± SD)**	31.1 ± 6.7	29.9 ± 5.7	0.01
**Waist to hip ratio (mean ± SD)**	1 ± 0.1	0.9 ± 0.1	<0.0001
**Systolic blood pressure, mm Hg (mean ± SD)**	134.1 ± 21.4	123.4 ± 17.3	<0.0001
**Diastolic blood pressure, mm Hg (mean ± SD)**	75 ± 12.1	72.5 ± 10.2	0.007
**Triglycerides, mg/dL (mean ± SD)**	147.9 ± 119.3	126 ± 87.5	<0.0001
**High density lipoprotein cholesterol, mg/dL (mean ± SD)**	49.1 ± 15	52.2 ± 15	0.0001
**Fasting glucose, mg/dL (mean ± SD)**	138.1 ± 72.1	108.3 ± 34.5	<0.0001
**Hypertension medication (%)**	54.8	31.8	<0.0001
**Diabetes medication (%)**	42.8	16.8	<0.0001
**Lipid-lowering medication (%)**	16.4	7.3	<0.0001
**eGFR, ml/min/1.73 m2 (mean ± SD)**	83.6 ± 28.9	102.1 ± 16.3	<0.0001
**Urinary albumin:creatinine ratio (mean ± SD)**	283.4 ± 792.5	5.6 ± 5	<0.0001

^a^Defined as UAC ratio ≥30 and/or eGFR <60 ml/min/1.73 m^2^ based on CKD-EPI creatinine-cystatin C equation without race.

^b^P-value from Wilcoxon rank-sum test for continuous variables or Chi-square test for categorical variables.

### Kidney traits and gut microbiome diversity

Kidney traits (eGFR [continuous, binary], UAC ratio [continuous, binary], and CKD) were not related to the Shannon diversity index in multivariable linear regression models adjusted for demographic and microbiome-related factors (Model 1), behavioral and socioeconomic factors (Model 2), and cardiometabolic factors (Model 3) (Supplementary Table 4). Continuous eGFR was significantly associated with overall gut microbiome composition, measured by the Jensen-Shannon Divergence (JSD) and generalized UniFrac distance, in multivariable permutational multivariate analysis of variance (PERMANOVA) models (from Model 3, JSD R-squared = 0.19%, *p* = 0.001; generalized UniFrac R-squared = 0.11%, *p* = 0.001) (Figure 1b; Supplementary Table 5). Other kidney traits (binary eGFR, continuous and binary UAC ratio, and CKD) were not associated with the JSD or generalized UniFrac distance upon full covariate adjustment (all *p* > 0.15) (Figure 1b; Supplementary Table 5). Consistently, only eGFR was significantly correlated with the first JSD principal coordinate (Figure 1c), with a visual shift along the first principal coordinate axis for participants with low eGFR (Figure 1d), but not for those with high UAC ratio (Figure 1e). In sensitivity analyses restricting to participants with none or little change in kidney function and damage over the past 6 y, continuous eGFR remained the only significant kidney trait predictor of the JSD (Supplementary Figure S2). Further, continuous eGFR remained a significant predictor of the JSD when excluding participants with low eGFR (<60 ml/min/1.73 m^2^) (R-squared = 0.14%, *p* = 0.006), suggesting that the finding is not solely driven by advanced CKD.

In stratified analysis of diabetes status, the UAC ratio (binary and continuous) and CKD were associated with significantly lower Shannon diversity index and altered overall gut microbiome composition measured by the JSD, only in participants without diabetes (all p-interaction <0.05) (Supplementary Table 6). While the association of continuous eGFR with overall microbiome composition was only significant in participants with diabetes, the interaction was not significant (p-interaction = 0.15) (Supplementary Table 6).

Based on the stronger relationships of continuous eGFR and UAC ratio with overall microbiome composition over the binary variables (Figure 1b), subsequent analyses focused on continuous eGFR and UAC ratio, as well as CKD.

### Kidney traits and gut microbiome species

**Figure 2.**
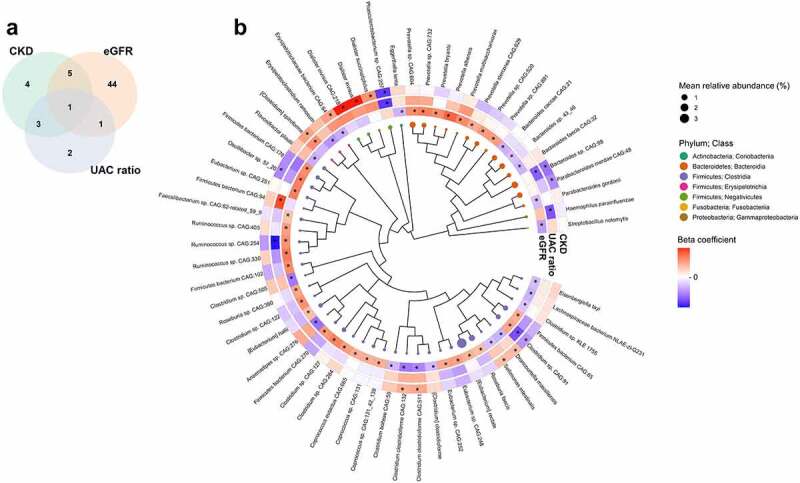
Gut microbiome species associated with kidney traits in the Hispanic Community Health Study/Study of Latinos (*N* = 2,438). (a) Venn diagram of unique and overlapping species associated with eGFR, UAC ratio, and CKD in ANCOM2 models at a detection level of 0.7 or above. ANCOM2 models were adjusted for age, sex, field center, Hispanic/Latino background, U.S. nativity, antibiotics use, Bristol stool type, income, educational attainment, cigarette smoking, alcohol use, AHEI2010, predicted sodium intake, report of low-sodium diet, predicted protein intake, report of high protein/low carb diet, protein supplement use, total physical activity, BMI, waist-to-hip ratio, systolic blood pressure, diastolic blood pressure, triglycerides, HDL cholesterol, fasting glucose, hypertension medication, diabetes medication, and lipid-lowering medication. (b) Phylogenetic tree of species associated with eGFR, UAC ratio, and CKD in ANCOM2 models described in (a). Node size reflects mean relative abundance, while node colors for species reflects taxonomic class. Effect size (beta) coefficients from multivariable linear regression of kidney traits on clr-transformed species abundance, adjusting for aforementioned covariates, are displayed in a circular heatmap around the tree. Beta coefficient legend key ranges from−0.015 to 0.015 for eGFR, −0.155 to 0.155 for log UAC ratio, and−0.81 to 0.81 for CKD. *Detection level ≥0.7 in ANCOM2 for a given kidney trait.

Of 1,177 species tested in Analysis of Composition of Microbiomes (ANCOM2), 51, 7, and 13 species were associated with eGFR, UAC ratio, and CKD, respectively, at a detection level ≥0.7 with full covariate adjustment (Figure 2a). Higher eGFR was associated with enrichment of eight species from genus *Prevotella*, and many species from class Clostridia within genera *Eubacterium*, *Clostridium*, *Roseburia*, and *Ruminococcus* (Figure 2b; Supplementary Table 7). Higher eGFR was also related to depletion of species in classes Erysipelotrichia, Clostridia, Coriobacteriia, and Fusobacteriia (Figure 2b; Supplementary Table 7). Higher UAC ratio was associated with lower abundance of *Clostridium sp. CAG:91*, *Ruminococcus sp. CAG:254* (both overlapping with eGFR), *Haemophilus parainfluenzae*, *Bacteroides sp. CAG:98*, and *Phascolarctobacterium sp. CAG:207*, and higher abundance of *[Clostridium] spiroforme* and *Firmicutes bacterium CAG:94* (Figure 2b; Supplementary Table 8). The majority of species associated with CKD were also related to eGFR and/or UAC ratio (Figure 2b; Supplementary Table 9). Associations of species with kidney traits were similar in a sensitivity analysis excluding participants with low eGFR (Suplementary Figure S3).

In stratified analysis of diabetes status, eGFR was associated with a greater number of species in participants with diabetes compared to those without diabetes, though effect estimates for eGFR-related species were similar for those with and without diabetes, with few significant interactions by diabetes status (Supplementary Figure S4a; Supplementary Table 10). In contrast, more species were associated with UAC ratio and CKD in participants without diabetes compared to those with diabetes, and effect estimates for UAC ratio-related species were not similar for participants with and without diabetes (Supplementary Figure S4a; Supplementary Table 10).

### Kidney traits and gut microbiome functions

**Figure 3.**
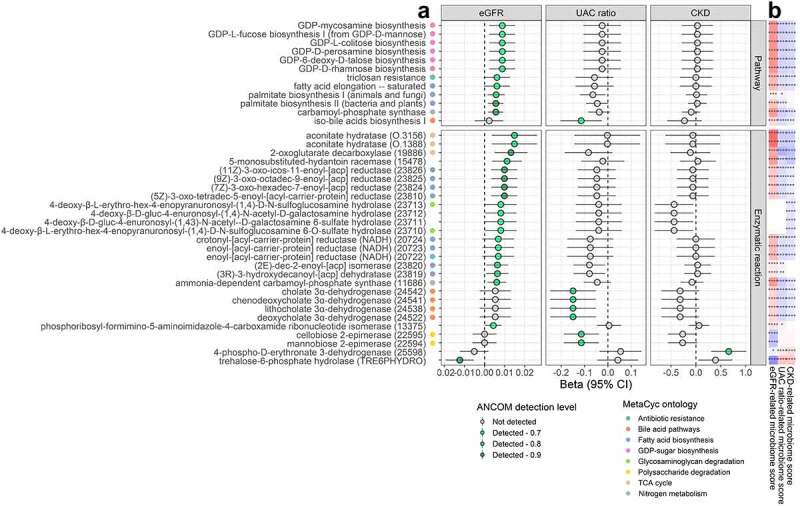
Gut microbiome functions associated with kidney traits in the Hispanic Community Health Study/Study of Latinos (*N* = 2,438). (a) We used ANCOM2 to identify MetaCyc pathways and enzymatic reactions for which abundance was associated with eGFR, UAC ratio, or CKD. ANCOM2 models were adjusted for age, sex, field center, Hispanic/Latino background, U.S. nativity, antibiotics use, Bristol stool type, income, educational attainment, cigarette smoking, alcohol use, AHEI2010, predicted sodium intake, report of low-sodium diet, predicted protein intake, report of high protein/low carb diet, protein supplement use, total physical activity, BMI, waist-to-hip ratio, systolic blood pressure, diastolic blood pressure, triglycerides, HDL cholesterol, fasting glucose, hypertension medication, diabetes medication, and lipid-lowering medication. For each pathway/reaction associated with at least one kidney trait in ANCOM2 (detection level ≥0.7), we show the estimated effect size (beta) and 95% confidence interval for eGFR (ml/min/1.73 m^2^), log UAC ratio, and CKD from multivariable linear regression models, with clr-transformed pathway/reaction abundance as outcomes, adjusting for same covariates as used in ANCOM2. Pathways/reaction names are annotated with colored circles according to their ontology in the MetaCyc database (only for categories with ≥2 constituents). (b) Spearman correlations of kidney trait-related microbiome scores and clr-transformed pathway/reaction abundance. Scores were derived by Z-score standardizing the clr-transformed abundance of species associated with a given kidney trait, followed by summing/subtracting species that were positively/negatively related to the trait.

Of 660 functional pathways and 1,067 enzymatic reactions tested in ANCOM2, 11 pathways and 20 reactions were associated with eGFR at a detection level ≥0.7 with full covariate adjustment (Figure 3a; Supplementary Tables 11–12). Higher eGFR was associated with higher abundance of GDP-sugar biosynthesis, fatty acid biosynthesis, nitrogen metabolism, glycosaminoglycan degradation, and TCA cycle functions (see Supplementary Table 13 for pathway/reaction ontology). The UAC ratio was associated with lower abundance of iso-bile acid synthesis and polysaccharide degradation functions (Figure 3a; Supplementary Tables 14–15). Lastly, CKD was associated with higher abundance of a D-erythronate degradation reaction (Figure 3a; Supplementary Tables 16–17). While different pathways and reactions were associated with kidney traits in participants with and without diabetes, few significant interactions were observed for these pathways and reactions by diabetes status (Supplementary Figure S4b-c; Supplementary Table 10).

Kidney trait-related microbiome scores, derived from species associated with eGFR, UAC ratio, or CKD, were significantly correlated with most kidney-trait related microbiome functions (Figure 3b). The eGFR-related microbiome score was most strongly positively correlated with abundance of aconitate hydratase enzymatic reactions (Figure 3b). Additionally, microbiome functions positively related to eGFR tended to positively correlate with eGFR-enriched species (Supplementary Figure S5). For example, carbamoyl-phosphate synthase and aconitate hydratase had strong positive correlations with eGFR-enriched *Prevotella* species, and fatty acid biosynthesis reactions were positively correlated with *[Eubacterium] rectale* (Supplementary Figure S5).

### Kidney trait-related microbiome features and serum metabolites

**Figure 4.**
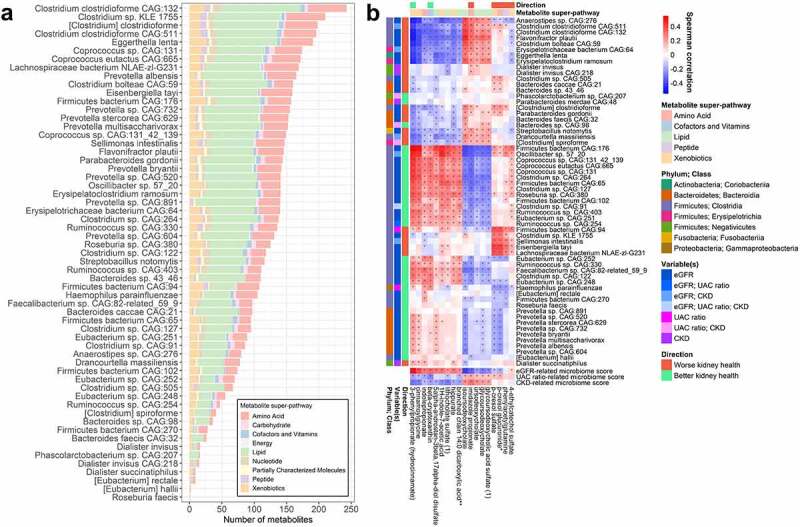
Serum metabolites are associated with kidney-related gut microbiome species (*N* = 700). (a) Number of serum metabolites (out of 773 named metabolites) associated with kidney trait-related species in Spearman correlation analysis at an FDR-adjusted p-value (q-value) <0.05. Species were clr-transformed and metabolites were inverse-normal transformed for analysis. Only species related to kidney traits in ANCOM2 analysis were included. (b) Spearman correlations of species and kidney trait-related microbiome scores with serum metabolites. Only metabolites that were correlated with at least 1 of these species (q < 0.05) with |r| ≥ 0.3 were included in the heatmap. Species are annotated on the side with taxonomic class, variable(s) they were associated with in ANCOM2, and direction of association with kidney health. Metabolites are annotated on the top with their super-pathway classification, and direction of correlation with eGFR (only if q < 0.05 for correlation with eGFR). *q < 0.05.

Abundance of *Clostridium clostridioforme CAG:132* was correlated with the greatest number of metabolites after FDR adjustment (243 out of 773 metabolites tested) (Figure 4a; Supplementary Table 18). When considering only the strongest correlations (Spearman |r| ≥ 0.3, q < 0.05), 18 metabolites were associated with at least 1 kidney trait-related species (Figure 4b); their peak areas according to binary eGFR, UAC ratio, and CKD status is shown in Supplementary Table 19. In general, gut microbiome species related to better kidney health were positively correlated with hydrocinnamate, cinnamoylglycine, indolepropionate, beta-cryptoxanthin, 4-ethylcatechol sulfate, 5alpha-androstan-3beta,17alpha-diol disulfate, 1 H-indole-7-acetic acid, lithocholate sulfate, hippurate, and branched chain 14:0 dicarboxylic acid (Figure 4b). Conversely, species related to worse kidney health were positively correlated with deoxycholic acid metabolites, imidazole propionate, and uremic toxins p-cresol sulfate, p-cresol glucuronide, and phenylacetylglutamine (Figure 4b). Correlations remained similar when further adjusting for age, sex, field center, eGFR, and UAC ratio (Supplementary Figure 6), and correlations also appeared similar in participants with and without diabetes (Supplementary Figure 7).

Aconitate hydratase and carbamoyl-phosphate synthase enzymatic reaction abundance, related to higher eGFR, were inversely correlated with p-cresol sulfate and p-cresol glucuronide (Supplementary Figure 8a). Other kidney trait-related functional pathways and reactions did not strongly correlate (|r| ≥ 0.3, q < 0.05) with serum metabolites, but metabolites with |r| ≥ 0.2 were similar to those correlated with microbiome species (Supplementary Figure 8b; Supplementary Table 20). Among these, microbial iso-bile acid biosynthesis functions were inversely correlated with deoxycholic acid metabolites (Supplementary Figure 8b).

### Serum metabolites and prospective changes in kidney traits

**Figure 5.**
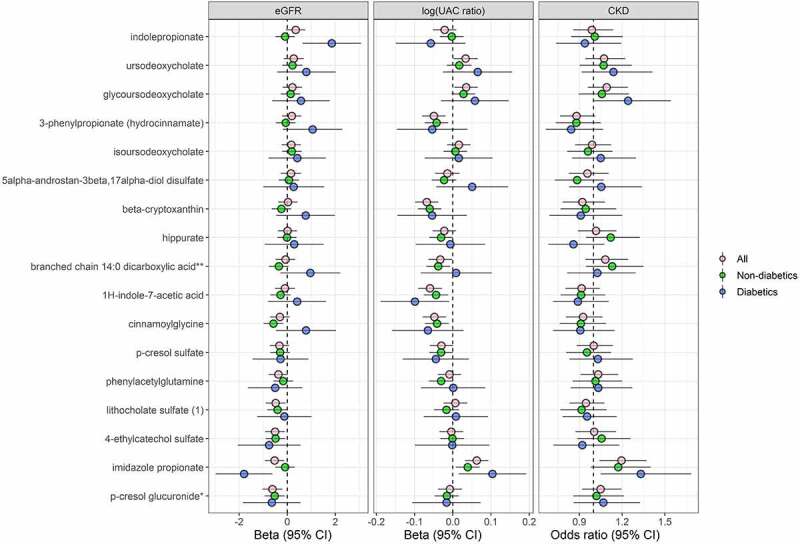
Serum metabolites associated with kidney-related gut microbiome species are predictors of kidney trait progression (*N* = 3,635). Prospective association of serum metabolites (selected from Figure 4b) with eGFR and UAC ratio progression, and incident CKD. For the continuous outcomes of eGFR and UAC ratio, multivariable linear mixed-effects regression models were used to estimate the effect of inverse-normal transformed metabolites at HCHS/SOL visit 1 on eGFR and UAC ratio progression from HCHS/SOL visit 1 to visit 2, adjusting for the following visit 1 covariates: age, sex, field center, Hispanic/Latino background, U.S. nativity, income, educational attainment, cigarette smoking, alcohol use, AHEI2010, predicted sodium intake, predicted protein intake, protein supplement use, total physical activity, BMI, waist-to-hip ratio, systolic blood pressure, diastolic blood pressure, triglycerides, HDL cholesterol, fasting glucose, hypertension medication, diabetes medication, and lipid-lowering medication. Betas are from the interaction of time x metabolite. For the binary outcome of incident CKD, multivariable logistic regression was used to estimate the effect of inverse-normal transformed metabolites at HCHS/SOL visit 1 on incidence of CKD at visit 2, adjusting for the visit 1 covariates listed above. Analyses were performed in all available participants (*n* = 3,635), non-diabetics (*n* = 2,979), and diabetics (*n* = 656).

For serum metabolites strongly correlated (|r| ≥ 0.3, q < 0.05) with kidney trait-related microbiome species (Figure 4b), we examined whether measures of these metabolites at HCHS/SOL visit 1 were associated with change in eGFR and UAC ratio, or with incidence of CKD, from visits 1 to 2 (over ~6 y). Metabolites associated with worsening kidney traits were lithocholate sulfate, 4-ethylcatechol sulfate, and p-cresol glucuronide (associated with reductions in eGFR), ursodeoxycholate and glycoursodeoxycholate (associated with increases in UAC ratio), and imidazole propionate (associated with reductions in eGFR, increases in UAC ratio, and incidence of CKD) (Figure 5). In contrast, hydrocinnamate, beta-cryptoxanthin, 1 H-indole-7-acetic acid, and cinnamoglycine were associated with reductions in UAC ratio over time (Figure 5). For some metabolites, associations differed in magnitude and/or statistical significance by diabetes status. For example, indolepropionate was associated with increases in eGFR only among participants with diabetes, and the association of imidazole propionate with worsening kidney traits was of greater magnitude in participants with diabetes (Figure 5). Findings were similar in the subset of participants randomly selected for metabolomics measurement (i.e., excluding participants selected based on kidney function decline), though some findings were attenuated, possibly due to reduced power (Supplementary Figure 9).

We also explored whether change in these serum metabolites over time (from visits 1 to 2) was associated with change in eGFR or UAC ratio over the same time period. We observed that increases in imidazole propionate and phenylacetylglutamine over time were associated with significant reductions in eGFR over time (*p* < 0.05), while changes in metabolites were not significantly related to change in UAC ratio over time (Supplementary Table 21).

## Discussion

In this large study of Hispanic/Latino adults, kidney function (eGFR) was significantly associated with overall gut microbiome composition and a wide range of species, while kidney damage (UAC ratio) was associated with lower gut microbiome diversity and altered composition only in participants without diabetes, suggesting that people with and without diabetes may have differences in gut microbiota involvement in kidney damage. Serum metabolites associated with kidney-related gut microbiome features (e.g., p-cresol glucuronide, imidazole propionate) were prospectively linked to changes in kidney function and damage over ~6 y, indicating that gut microbiota may play a role in CKD progression.

A number of previous studies observed depletion of taxa from *Prevotella*, *Faecalibacterium*, *Roseburia*, *Coprococcus*, and *Eubacterium* in patients with CKD compared to healthy controls^16–26^, similar to our findings of these taxa being related to higher eGFR. Many of these taxa are known SCFA producers, though we did not have measures of serum or stool SCFAs in this study to confirm their role. We also observed that microbial polysaccharide degradation functions (e.g., mannobiose and cellobiose 2-epimerase) were inversely related to the UAC ratio. These findings agree with evidence from other studies that SCFAs are depleted in stool and serum of CKD patients compared to healthy controls^27^, and that SCFAs ameliorate kidney injury^28^. SCFAs may protect the kidneys via several mechanisms, including reducing inflammation, oxidative stress, and pro-fibrotic factors in kidney cells^13^. Microbial functions related to synthesis of long-chain fatty acids, such as palmitate and oleate, were associated with higher eGFR. This was unexpected, since long-chain fatty acids accumulate in CKD and may induce renal injury^29^. We also found microbial ammonia-dependent carbamoyl-phosphate synthase was associated with higher eGFR, suggesting that ammonia utilization by gut bacteria^30^ may be beneficial for kidney health.

Our results of serum metabolites associated with kidney-trait related species suggest other microbial products which may preserve kidney function and/or reflect better kidney health. Diet-derived compounds, including hydrocinnamate and cinnamoglycine (from cinnamon) and beta-cryptoxanthin (from fruit), were positively correlated with species related to better kidney health, which may reflect the protective effect of healthy diet on incidence and progression of CKD^31,32^. In support of this, dietary fiber intake assessed at HCHS/SOL visit 1 was positively correlated with such diet-derived metabolites at visit 2, as well as with eGFR and species related to better kidney health (Supplementary Table 22). These metabolites were prospectively associated with reductions in the UAC ratio over time, suggesting they may prevent kidney damage or promote kidney structural repair. Indolepropionate, a bacterial product of tryptophan metabolism, was also positively correlated with species related to better kidney health. Indolepropionate was related to lower incidence of diabetes in a large multi-cohort analysis^33^ and also found to be lower in CKD patients vs. controls and associated with slower declines in kidney function^34^. We observed that indolepropionate was associated with increases in eGFR over time only among those with diabetes, indicating it may be important in diabetic kidney disease prevention.

In contrast, some microbial products may further CKD progression. Serum imidazole propionate, which was positively associated with species related to lower eGFR, is a microbial metabolite elevated in diabetes, shown to impair insulin signaling^35^. Consistently, we found that imidazole propionate was associated with prospective declines in eGFR and increases in UAC ratio, most prominently among participants with diabetes. Serum levels of microbially produced secondary bile acid deoxycholic acid have been associated with prospective CKD progression^36^ and coronary artery calcification in CKD^37^. We observed that species related to lower eGFR, including *Flavonifractor plautii* and *Eggerthella lenta*, were positively correlated with serum deoxycholic acid metabolites, while iso-bile acid biosynthesis functions (related to lower UAC ratio) were inversely correlated with deoxycholic acid metabolites. The iso-bile acid pathway is an oxidative pathway for bile acids that can detoxify deoxycholic acid^38^. Further, we found that deoxycholic acid metabolites were prospectively associated with increases in UAC ratio over time. Taken together, these results suggest that gut bacteria may promote kidney damage through synthesis of deoxycholic acid and/or insufficient conversion of deoxycholic acid to iso-bile acids. In agreement with these observations, other studies have observed enrichment of *Flavonifractor plautii* and *Eggerthella lenta* in CKD^23^, as well as enrichment of microbial secondary bile acid pathways in CKD^23,24^.

Microbially produced protein-bound uremic toxins are hypothesized as the primary mechanism for microbial involvement in CKD progression and morbidity. These include indoles (e.g., indoxyl sulfate), phenols (e.g., p-cresol sulfate, p-cresol glucuronide, phenylacetylglutamine), and hippurate^39,40^. Some studies found greater abundance of p-cresol and indoxyl sulfate-producing gut bacteria in CKD patients^11,23^, though one study suggests that the rate of bacterial generation of these toxins is not influenced by kidney function^41^. In our study, serum p-cresol sulfate, p-cresol glucuronide, and phenylacetylglutamine were positively correlated with some species related to worse kidney health, such as *Eisenenbergiella tayi* and *Sellimonas intestinalis*. Similar correlations, albeit slightly weaker, were observed for indoxyl sulfate (Supplementary Table 18), but serum hippurate was positively correlated with species related to better kidney health. In our prospective analysis, p-cresol glucuronide was associated with reductions in eGFR over time, confirming potential involvement of microbially produced uremic toxins in kidney disease.

Interestingly, despite the hypothesized contribution of uremia to gut microbiome dysbiosis in CKD^8,9^, in our study serum urea was only weakly associated with a few kidney trait-related species (Supplementary Table 18), suggesting that urea is not a primary driver of our findings. This may be because cases of CKD in our study were mostly mild CKD, thus serum urea is unlikely to be elevated enough to indicate uremia (though we did not have absolute measures of blood urea nitrogen in this study).

Associations of kidney traits with the gut microbiome differed by diabetes status. In participants without diabetes, higher UAC ratio was associated with reduced gut microbiome diversity and altered overall composition, while eGFR was not. In participants with diabetes, eGFR was a significant predictor of gut microbiome overall composition, while UAC ratio was not. While eGFR-microbiome relationships did not have statistically significant heterogeneity by diabetes status, the interaction of UAC ratio–microbiome relationships was significant. Diabetic and non-diabetic kidney disease develops with different pathological mechanisms. In diabetic kidney disease, or diabetic nephropathy, diabetes is the sole cause, with hyperglycemia being the central upstream driver^15^. Non-diabetic kidney disease can arise from hereditary or acquired causes, including poor nephron endowment, obesity, pregnancy, and injury- or aging-related nephron loss^15^. Without renal biopsy, etiology of CKD in people with diabetes (i.e., diabetic nephropathy or CKD coincident with diabetes) cannot be determined, as is the case in our study. Nevertheless, hyperglycemia is likely a contributing factor to CKD in people with diabetes regardless of etiology^15^. A number of studies have examined differences in the gut microbiome for people with diabetic kidney disease, diabetes without kidney disease, and healthy controls^42^, but little is known regarding gut microbiome associations with diabetic vs. non-diabetic kidney disease. Since we observed kidney damage was only related to the gut microbiome in participants without diabetes, this may suggest kidney damage in people with diabetes is attributed primarily to hyperglycemia and less to other factors (i.e., in a “sufficient causes” framework^43^, hyperglycemia may be a sufficient cause of kidney damage, precluding observation of an association with the gut microbiome). In accordance, fasting glucose was significantly correlated with the UAC ratio in participants with diabetes (Spearman *r* = 0.24, *p* < 0.0001), but not without diabetes (*r* = 0.00, *p* = 0.87). Alternatively, since sample size was lower in participants with diabetes, we may have had insufficient power to observe a relationship of the gut microbiome with kidney damage. Additional studies are needed to validate gut microbiome and kidney relationships in people with and without diabetes.

Our study was strengthened by large sample size, thorough control for potential confounders, analysis of continuous eGFR and UAC ratio across a wide continuum, and prospective analysis of microbiome-related metabolites with changes in kidney health. Our study also faced several limitations. CKD is usually defined with sustained low eGFR or albuminuria over a period of 3 months, but we relied on one-time measures. Our analysis of the gut microbiome and kidney traits was cross-sectional, limiting temporal inferences, though we did conduct a prospective analysis of microbiome-related metabolites with changes in kidney traits. We did not have measures of SCFAs to correlate with kidney-related species and functions. Finally, our study was performed solely in U.S. Hispanics/Latinos, which may limit generalizability.

In summary, our results suggest that the gut microbiome associated with better kidney health is characterized by higher abundance of *Prevotella* and Clostridia species known to produce SCFAs, while gut microbiota associated with poor kidney health may be involved in production of imidazole propionate, deoxycholic acid metabolites, and uremic toxins. Imidazole propionate, deoxycholic acid metabolites, and p-cresol glucuronide were associated with prospective reductions in eGFR and/or increases in the UAC ratio over ~6 y, suggesting their involvement in CKD progression and morbidity. Large prospective studies of the gut microbiome, microbial metabolites, and CKD progression are warranted to validate these results, followed by experimental models to support a causal relationship of the gut microbiome with CKD. Given the modifiable nature of the gut microbiome, there is promising therapeutic potential in altering the gut microbiome to prevent CKD progression^40^.

## Methods

### Study cohort

The Hispanic Community Health Study/Study of Latinos (HCHS/SOL) is a prospective, population-based cohort study of 16,415 Hispanic/Latino adults (ages 18–74 y at the time of recruitment during visit 1 [2008–2011]) who were selected using a multi-stage probability sampling design from randomly sampled census block areas within four U.S. communities (Chicago, IL; Miami, FL; Bronx, NY; San Diego, CA)^44,45^. The HCHS/SOL Gut Origins of Latino Diabetes ancillary study^46^ was conducted to examine the role of gut microbiome composition on diabetes and other outcomes, enrolling ~3,000 participants from the HCHS/SOL approximately concurrent with the second in-person HCHS/SOL visit cycle (visit 2, 2014–2017). For the cross-sectional analyses at visit 2 (Figure 1a), we excluded participants with prevalent cancer or CVD at visit 2, currently on kidney dialysis at visit 2, missing measures of serum creatinine, cystatin C, or UAC ratio at visit 2, or with <100K sequence reads in their microbiome sample (Supplementary Figure S1). For the prospective analysis from visits 1 to 2 (Figure 1a), we excluded participants with prevalent cancer, CVD, or CKD at visit 1, currently on kidney dialysis at visit 1, or missing measures of serum creatinine, cystatin C, or UAC ratio at visits 1 or 2 (Supplementary Figure S1). The study was conducted with the approval of the Institutional Review Boards (IRBs) of the five participating universities in HCHS/SOL. Written informed consent was provided by all study participants.

### Kidney trait definitions

Estimated glomerular filtration rate (eGFR) was calculated from serum creatinine and cystatin C using the new CKD-EPI creatinine-cystatin C equation without race^47^, as recommended by the National Kidney Foundation (NKF) and the American Society of Nephrology^48^. eGFR was considered as a continuous variable and a binary variable, with eGFR <60 ml/min/1.73 m^2^ indicating low eGFR. Kidney damage was assessed using the urinary albumin:creatinine (UAC) ratio, which was also considered as a continuous variable and a binary variable, with UAC ratio ≥30 indicating albuminuria. Chronic kidney disease (CKD) was defined as UAC ratio ≥30 and/or eGFR <60 ml/min/1.73 m^2^. Stages of CKD were defined using the standard NKF definition: Stage 1, eGFR ≥90 ml/min/1.73 m^2^ and albuminuria; Stage 2, eGFR 60–89 ml/min/1.73 m^2^ and albuminuria; Stage 3, eGFR 30–59 ml/min/1.73 m^2^; Stage 4, eGFR 15–29 ml/min/1.73 m^2^; Stage 5, eGFR <15 ml/min/1.73 m^2^.

### Assessment of cardiometabolic traits

Using an automatic sphygmomanometer, three seated blood pressure measures were obtained for each participant after a 5-minute rest period and means of the second and third measurement were used to derive systolic blood pressure and diastolic blood pressure^49^. Centralized laboratory tests included blood glucose, insulin, hemoglobin A1c (HbA1c), triglycerides, and total, high-density lipoprotein (HDL), and low-density lipoprotein (LDL) cholesterol, all measured after overnight fast^50^. Diabetes was defined based on meeting the American Diabetes Association lab criteria for diabetes (fasting glucose, glucose post-oral glucose tolerance test, or HbA1c) or self-report of anti-diabetic medication.

### Covariate data

Participant characteristics were included for statistical adjustment in our analysis, based on known or suspected relationships with CKD and/or the gut microbiome. These variables were age (continuous), sex (male, female), field center (Chicago, Miami, Bronx, San Diego), Hispanic/Latino background (Dominican, Central American, South American, Cuban, Mexican, Puerto Rican, more than one heritage/other/missing), U.S. nativity (born in 50 U.S. states/DC or a U.S. territory, foreign born), antibiotic use in last 6 months (yes, no), Bristol stool type (8 categories), income (<$30,000, ≥30,000, missing), educational attainment (less than high school, high school or equivalent, greater than high school, missing), cigarette smoking (never, former, current), alcohol use (nondrinker, low-level use, high-level use), the Alternative Healthy Eating Index 2010 (AHEI2010; continuous), predicted sodium intake based on the NCI method (continuous), report of low-sodium diet (yes, no), predicted protein intake based on the NCI method (continuous), report of high protein/low carbohydrate diet (yes, no), protein supplement use (yes, no), total physical activity based on the Global Physical Activity Questionnaire (GPAQ; continuous), BMI (continuous), waist-to-hip ratio (continuous), systolic blood pressure (continuous), diastolic blood pressure (continuous), triglycerides (continuous), HDL cholesterol (continuous), fasting glucose (continuous), anti-hypertensive medication (yes, no), anti-diabetic medication (yes, no), lipid-lowering medication (yes, no). For the cross-sectional analyses at visit 2, all covariates were based on visit 2 data, except AHEI10, predicted sodium intake, and predicted protein intake which were based on visit 1 data (since dietary recalls were only collected at visit 1). For the prospective analysis from visits 1 to 2, all covariates were based on visit 1 data. Missing covariate data were imputed at the median and mode for continuous and categorical variables, respectively, with the exception of categorical variables with >1% missing, for which a missing category was created.

### Microbiome measurement

Stool samples were collected by participants at home using stool collection kits, as described previously^46^. Shallow shotgun sequencing was conducted in the Knight laboratory at the University of California San Diego^51^, as previously described in HCHS/SOL^52^. Briefly, DNA was extracted from fecal samples following the Earth Microbiome Project protocol^53^. Adapters and barcode indices were added following the iTru adapter protocol^54^, and the resulting libraries were purified, quantified, and normalized for sequencing on Illumina NovaSeq.

### Microbiome bioinformatics processing

FASTQ sequence reads were processed using the standard shotgun sequencing pipeline in Qiita. Briefly, per sample sequence adapters were removed via fastp, and sequence reads mapping to the human genome were filtered via minimap2. The sequence reads were then aligned against the WolR1 reference database of bacterial and archaeal genomes using Woltka with the Bowtie2 aligner^55^, to generate an operational genomic unit (OGU) table and a gene table. The sequence alignments were also classified at the species taxonomic rank, while functional profiles were obtained by collapsing the gene table into MetaCyc enzymatic reactions and functional pathways. Indices of α-diversity (Shannon diversity index) and β-diversity (Jensen-Shannon Divergence, generalized UniFrac) were calculated from the OGU table using “vegan,” “phyloseq,” and “GUniFrac” packages in R^56–58^.

### Metabolomics measurement

A subset of 825 participants had available metabolomics profiling of visit 2 fasting serum samples; these participants were selected for metabolomics profiling based on availability of gut microbiome samples collected within 30 d of visit 2. After restricting by our exclusion criteria (Suplementary Figure S1), 700 participants remained for cross-sectional metabolomics analysis at visit 2. Additionally, a subset of 6,180 participants had available metabolomics profiling of visit 1 fasting serum samples; 3,978 were a random subsample, while the remainder were selected based on participation in the echocardiographic ancillary study of HCHS/SOL or based on decline in eGFR from visits 1 to 2 (the random subsample and pre-selected samples were measured in different batches, “batch 1” and “batch 2,” which are pooled here). After restricting by our exclusion criteria (Suplementary Figure S1), 3,635 participants remained for prospective analysis of visit 1 metabolomics data. Using the discoveryHD4 platform at Metabolon Inc., quantification of serum metabolites was achieved by using an untargeted LC-MS-based metabolomic quantification protocol, as previously described^59^. We imputed values below detection as half the minimum value per metabolite.

### Statistical analysis

#### General principles

An overview of the analyses is provided in Figure 1a. The kidney traits considered in the statistical analyses were eGFR (continuous or binary), UAC ratio (continuous or binary), and CKD (binary). The continuous UAC ratio was log-transformed for all analyses due to a heavy right skew. All analyses were conducted in R version 3.6.3.

#### Cross-sectional analyses of the gut microbiome and kidney traits

We considered nested models to serially adjust for potential confounders, based on known factors related to CKD, and to the gut microbiome in this cohort^46^. Model 1 (demographic and microbiome-related factor model) adjusted for age, sex, field center, Hispanic/Latino background, U.S. nativity, antibiotics use, and Bristol stool type. Model 2 (socioeconomic and behavioral model) adjusted for Model 1 covariates plus income, educational attainment, cigarette smoking, alcohol use, AHEI2010, predicted sodium intake, report of low-sodium diet, predicted protein intake, report of high protein/low carbohydrate diet, protein supplement use, and total physical activity. Model 3 (cardiometabolic model) adjusted for Model 2 covariates plus BMI, waist-to-hip ratio, systolic blood pressure, diastolic blood pressure, triglycerides, HDL cholesterol, fasting glucose, hypertension medication, diabetes medication, and lipid-lowering medication.

1. *Within-subject (α-) and between-subject (β-) diversity*. Multivariable linear regression was used to examine the association of kidney traits (predictors) with the Shannon diversity index (outcome), adjusting for covariates. Permutational multivariate analysis of variance (PERMANOVA) was used to assess the association of kidney traits with overall microbiome composition, as measured by the Jensen-Shannon Divergence and generalized UniFrac distance, adjusting for covariates. A p-value <0.05 was considered significant in diversity analyses.
2. *Species and metagenomic pathways/enzymatic reactions*. Microbial species and MetaCyc functional pathways and enzymatic reactions were analyzed in two stages: first using the Analysis of Composition of Microbiomes (ANCOM2) method^60^, followed by confirmatory multivariable linear regression, described below. ANCOM2 was used to detect species, pathways, and reactions for which abundance was related to kidney traits, adjusting for covariates. We controlled the false discovery rate (FDR) at 5% and excluded species, pathways, or reactions from testing if they were present in <20% of the participants. An ANCOM2 detection level ≥0.7 was considered significant – this level indicates that the ratios of the species, pathway, or reaction to at least 70% of other species, pathways, or reactions were detected to be significantly associated (FDR q < 0.05) with a kidney trait. To assess the direction and magnitude of the associations, we constructed multivariable linear regression models, with centered log ratio (clr)-transformed species/pathway/reaction abundance as outcomes, and kidney traits as the main predictors, adjusting for covariates.
3. *Kidney trait-related microbiome scores*. We developed kidney trait-related microbiome scores based on species associated with eGFR, UAC ratio, or CKD, to relate with gut microbiome functional pathways/enzymatic reactions and serum metabolites. First, clr-transformed abundance of species associated with a given kidney trait in ANCOM2 (detection level ≥0.7) was Z-score standardized to give equal weight to each species; then, those species positively related to the kidney trait were summed while species negatively related to the kidney trait were subtracted within each participant to derive the score.

#### Cross-sectional analysis of serum metabolites and kidney-related microbiome features

Metabolite concentrations were inverse-normal transformed for analysis. Only named metabolites with <20% missing were considered, totaling 773 metabolites. We examined unadjusted and partial Spearman correlations (adjusting for age, sex, field center, eGFR, and UAC ratio) of metabolites with kidney-related gut microbiome species, functional pathways, and enzymatic reaction clr-transformed abundance. We similarly examined correlations of serum metabolites with kidney-trait related microbiome scores. For each microbiome feature, we considered a metabolite with FDR-adjusted p-value (q-value) <0.05 as a significantly correlated metabolite.

#### Prospective analysis of serum metabolites and kidney trait progression

For specific metabolites strongly correlated (Spearman |r| ≥ 0.3) with kidney trait-related microbiome species, we examined associations with eGFR and UAC ratio progression and incident CKD. For the continuous outcomes of eGFR and UAC ratio, multivariable linear mixed-effects regression models with a random intercept were used to estimate the effect of inverse-normal transformed metabolites at visit 1 on eGFR and UAC ratio progression from visit 1 to visit 2, adjusting for the following visit 1 covariates: age, sex, field center, Hispanic/Latino background, U.S. nativity, income, educational attainment, cigarette smoking, alcohol use, AHEI2010, predicted sodium intake, predicted protein intake, protein supplement use, total physical activity, BMI, waist-to-hip ratio, systolic blood pressure, diastolic blood pressure, triglycerides, HDL cholesterol, fasting glucose, hypertension medication, diabetes medication, and lipid-lowering medication. The effect of interest was the interaction of time × metabolite. For the binary outcome of incident CKD, multivariable logistic regression was used to estimate the effect of inverse-normal transformed metabolites at visit 1 on incidence of CKD at visit 2, adjusting for the visit 1 covariates listed above. For this analysis based on pre-specified predictors and outcomes, *p* < 0.05 was considered significant.

#### Sensitivity and stratified analyses

We conducted several sensitivity analyses to confirm our findings. For the cross-sectional analysis of kidney traits and gut microbiome β-diversity, we performed a sensitivity analysis restricting to participants with either no change in binary eGFR, UAC ratio, and CKD status (*n* = 2,042), or <10% change in eGFR (*n* = 1,614), from HCHS/SOL visits 1 to 2 (approximately 6 y apart) – this was to confirm that findings remained similar in participants who had consistent kidney function over the past 6 y. Additionally, we performed a sensitivity analysis excluding participants with low eGFR (<60 ml/min/1.73 m^2^), to confirm that findings were not solely driven by those with advanced stages of CKD. Lastly, we performed stratified analyses to determine whether associations of the gut microbiome and kidney traits differ by diabetes status and tested for interaction of diabetes and kidney traits on the gut microbiome using cross-product terms.

## Supplementary Material

Supplemental Material

## Data Availability

HCHS/SOL data are archived at the National Institutes of Health repositories dbGap and BIOLINCC. Sequence data from the samples described in this study is deposited in QIITA (study ID 11666). HCHS/SOL has established a process for the scientific community to apply for access to participant data and materials, including the metabolomics data used herein, with such requests reviewed by the project’s Steering Committee. These policies are described at https://sites.cscc.unc.edu/hchs/.

## List of abbreviations

AHEI2010, Alternative Health Eating Index 2010; ANCOM, Analysis of Composition of Microbiomes; CKD, chronic kidney disease; CLR, centered log-ratio; CVD, cardiovascular disease; eGFR, estimated glomerular filtration rate; FDR, false discovery rate; HCHS/SOL, Hispanic Community Health Study/Study of Latinos; HDL, high-density lipoprotein; JSD, Jensen-Shannon Divergence; PERMANOVA, permutational multivariate analysis of variance; SCFA, short-chain fatty acid; UAC ratio, urinary albumin-to-creatinine ratio.
