# Supplementary material for: Association of the gut microbiome with kidney function and damage in the Hispanic Community Health Study/Study of Latinos (HCHS/SOL)
Source: Gut Microbes. 2023 Mar 7;15(1):2186685. doi: 10.1080/19490976.2023.2186685 (PMC10012940; doi:10.1080/19490976.2023.2186685)
Supplement: Supplemental Material [file KGMI_A_2186685_SM5435.zip › SUPPLEMENTARY FIGURES_1_17_23.docx]

**SUPPLEMENTARY FIGURES**

**Association of the gut microbiome with kidney function and damage in the Hispanic Community Health Study/Study of Latinos (HCHS/SOL)**

Brandilyn A. Peters, Qibin Qi, Mykhaylo Usyk, Martha L. Daviglus, Jianwen Cai, Nora Franceschini, James P. Lash, Marc D. Gellman, Bing Yu, Eric Boerwinkle, Rob Knight, Robert D. Burk, Robert C. Kaplan


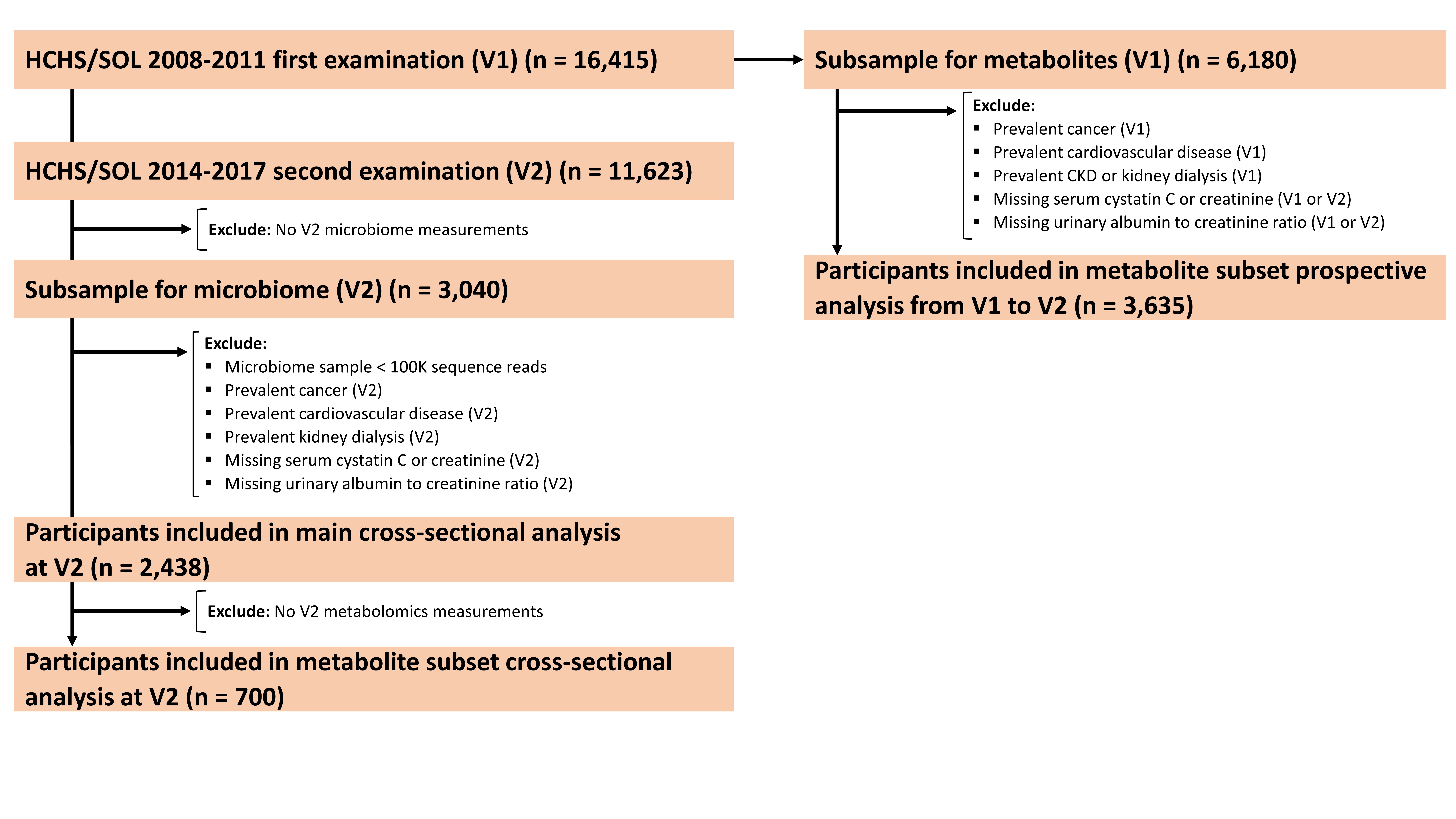


**Supplementary Figure 1.** Flow chart of participants included in the analyses.


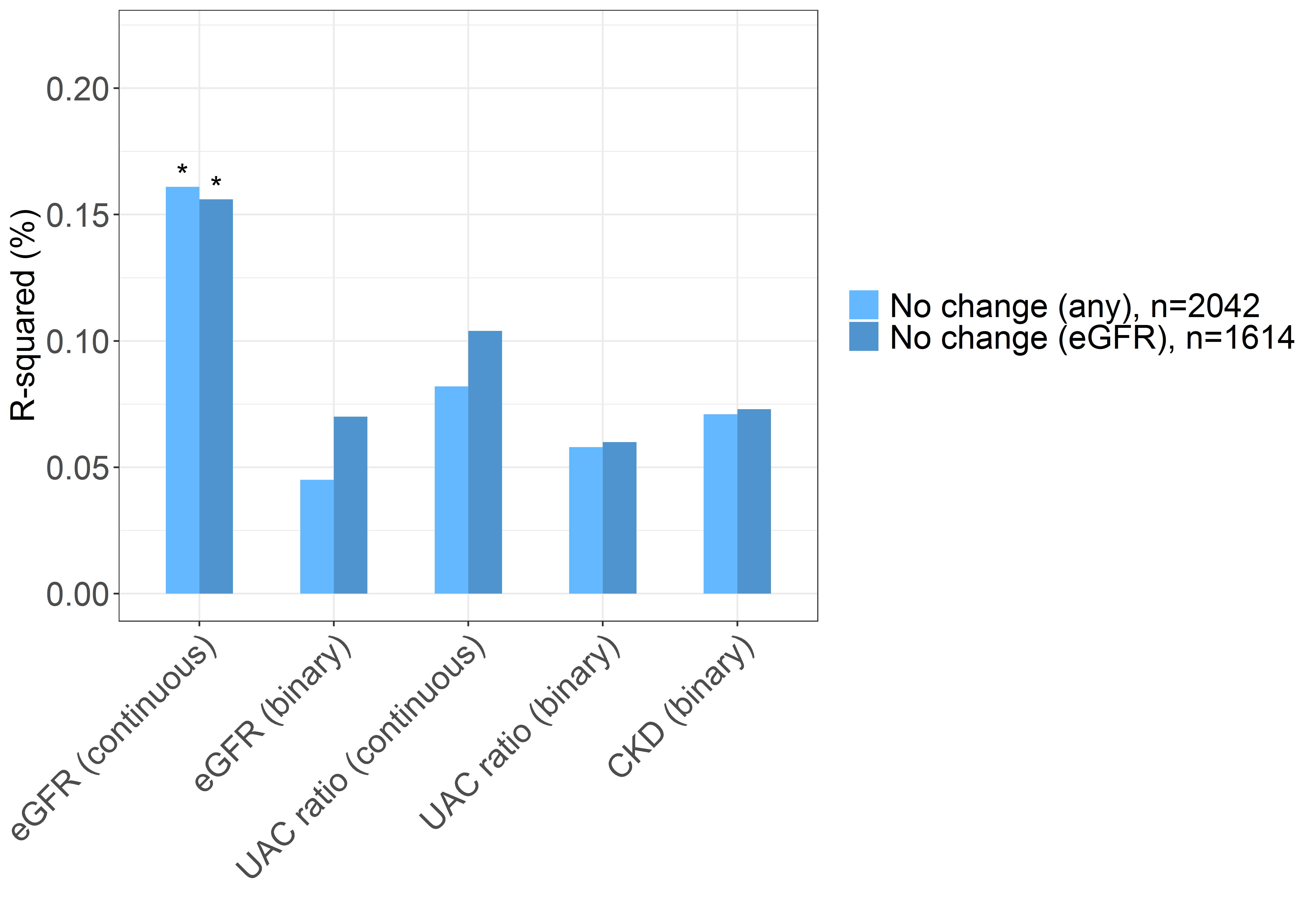


**Supplementary Figure 2.** Barplot shows R-squared (%) for kidney traits from PERMANOVA models of the Jensen-Shannon Divergence, among participants with stable kidney status (no change in binary CKD, binary UAC ratio, or binary eGFR status) or stable eGFR (change <10%) from the 1^st^ to 2^nd^ HCHS/SOL study visits. Each kidney trait (predictor) was assessed in a separate model, adjusted for age, sex, field center, Hispanic/Latino background, U.S. nativity, antibiotics use, Bristol stool type, income, educational attainment, cigarette smoking, alcohol use, AHEI2010, predicted sodium intake, report of low-sodium diet, predicted protein intake, report of high protein/low carb diet, protein supplement use, total physical activity, BMI, waist-to-hip ratio, systolic blood pressure, diastolic blood pressure, triglycerides, HDL cholesterol, fasting glucose, hypertension medication, diabetes medication, and lipid-lowering medication. *p<0.05, **p<0.01.


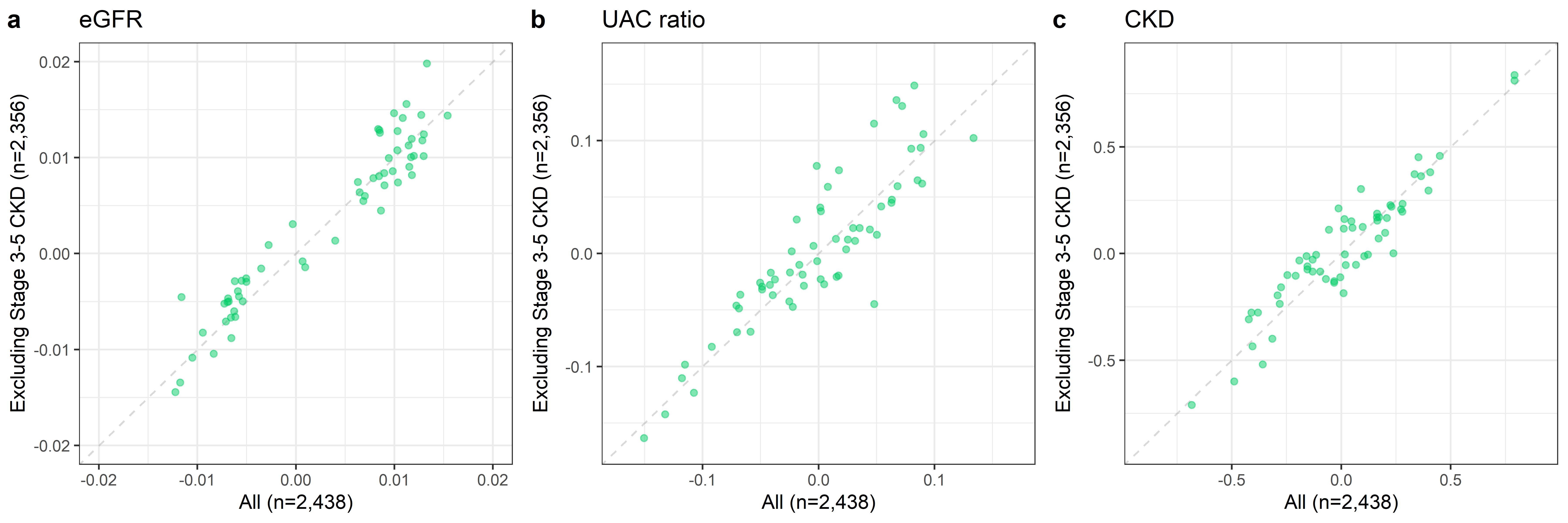


**Supplementary Figure 3.** Correlation of effect size (beta) for 56 kidney trait-related species with (a) eGFR, (b) log UAC ratio, and (c) CKD, among all participants (x-axis) vs. excluding those with Stage 3-5 CKD (y-axis). Effect size (beta) coefficients from multivariable linear regression of kidney traits on clr-transformed species abundance, adjusting for age, sex, field center, Hispanic/Latino background, U.S. nativity, antibiotics use, Bristol stool type, income, educational attainment, cigarette smoking, alcohol use, AHEI2010, predicted sodium intake, report of low-sodium diet, predicted protein intake, report of high protein/low carb diet, protein supplement use, total physical activity, BMI, waist-to-hip ratio, systolic blood pressure, diastolic blood pressure, triglycerides, HDL cholesterol, fasting glucose, hypertension medication, diabetes medication, and lipid-lowering medication.


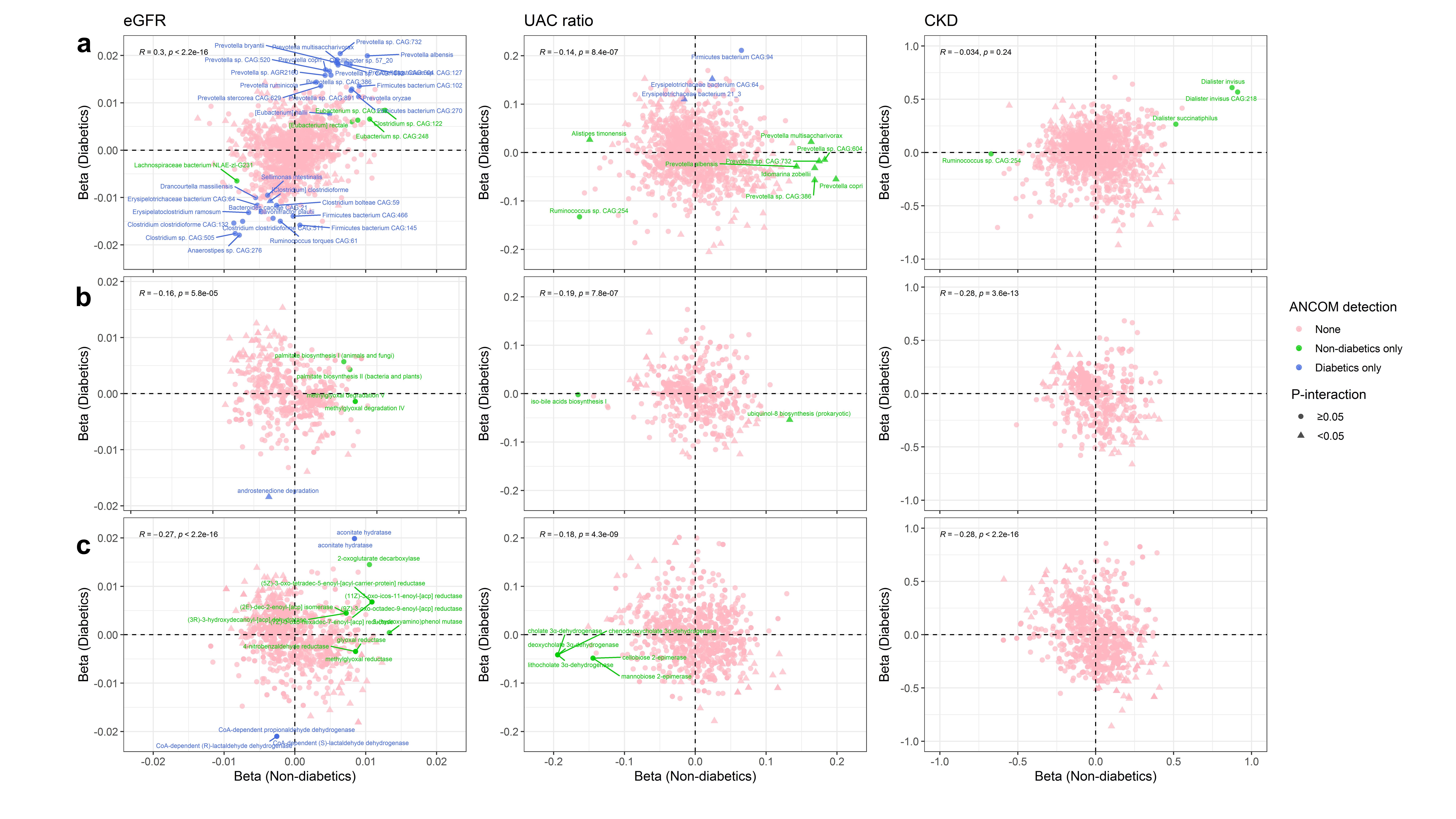


**Supplementary Figure 4.** Correlation of effect size (beta) for (a) species, (b) functional pathways, and (c) enzymatic reactions with kidney traits, among non-diabetics n=1740 (x-axis) vs. diabetics n=698 (y-axis). Effect size (beta) coefficients from multivariable linear regression of kidney traits on clr-transformed species, pathway, or reaction abundance, adjusting for age, sex, field center, Hispanic/Latino background, U.S. nativity, antibiotics use, Bristol stool type, income, educational attainment, cigarette smoking, alcohol use, AHEI2010, predicted sodium intake, report of low-sodium diet, predicted protein intake, report of high protein/low carb diet, protein supplement use, total physical activity, BMI, waist-to-hip ratio, systolic blood pressure, diastolic blood pressure, triglycerides, HDL cholesterol, fasting glucose, hypertension medication, diabetes medication, and lipid-lowering medication. Microbiome features with significant ANCOM2 detection (detection level≥0.7) in either non-diabetics or diabetics are indicated in text on the plots. Features with a significant interaction (diabetes status x kidney trait) are indicated with triangles.





**Supplementary Figure 5.** Spearman correlations among kidney trait-related species, functional pathways, and enzymatic reaction clr-transformed abundance (n=2,438). Microbiome features are annotated with taxonomic class (species only) or type (species, pathway, or enzymatic reaction), variable(s) they were associated with in ANCOM2, and direction of association with kidney health. *p<0.05, **p<0.01, ***p<0.001, ****p<0.0001.





**Supplementary Figure 6.** Partial Spearman correlations of clr-transformed species abundance and kidney trait-related microbiome scores with inverse-normal transformed serum metabolites, adjusting for age, sex, field center, eGFR, and UAC ratio (n=700). Metabolites included are the same as in Figure 4b of main manuscript. Species are annotated on the side with taxonomic class, variable(s) they were associated with in ANCOM2, and direction of association with kidney health. Metabolites are annotated on the top with their super-pathway classification, and direction of correlation with eGFR (only if q<0.05 for correlation with eGFR). *q<0.05.


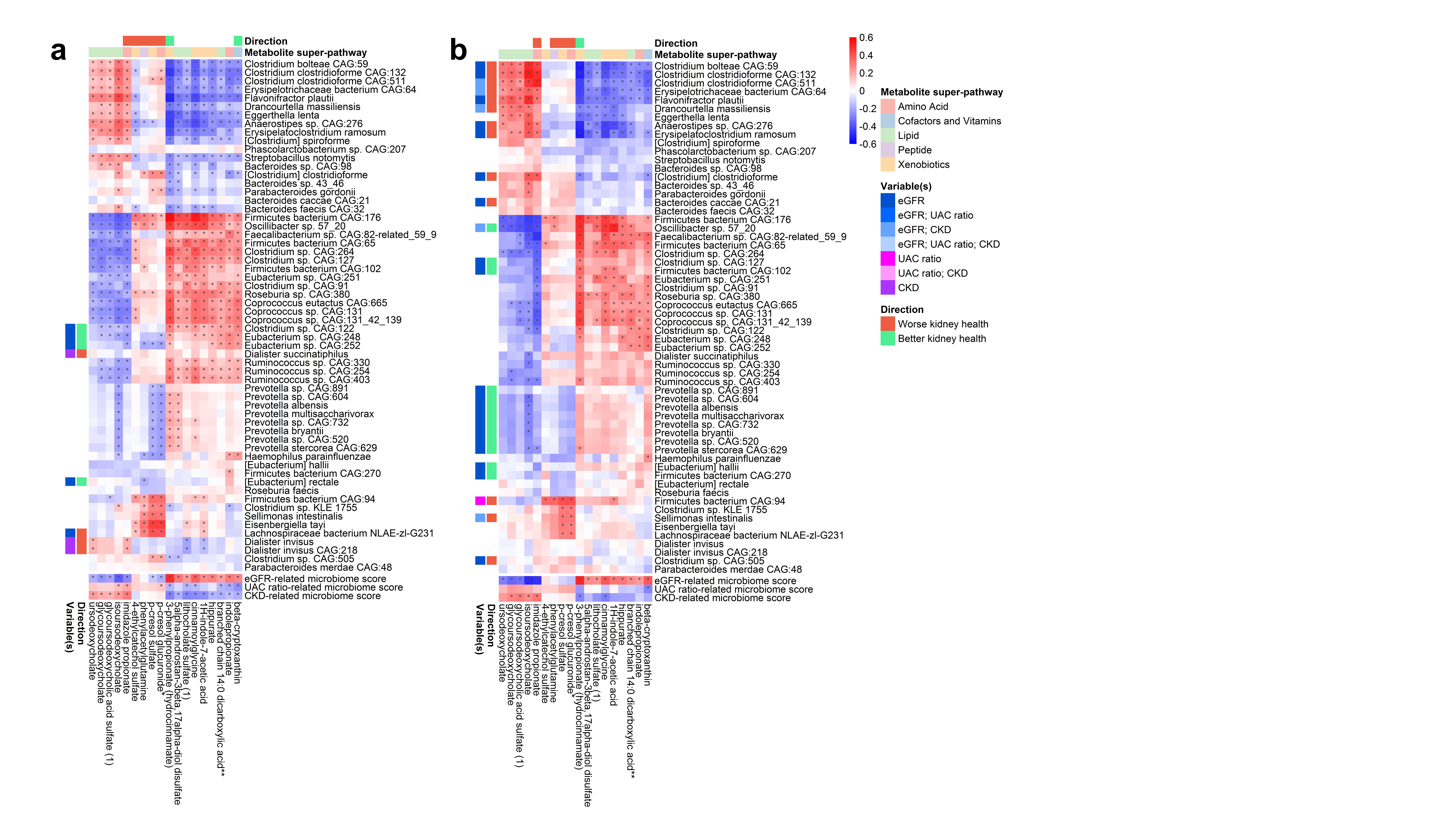


**Supplementary Figure 7.** Spearman correlations of clr-transformed species abundance and kidney trait-related microbiome scores with inverse-normal transformed serum metabolites, in (a) non-diabetics (n=507) and (b) diabetics (n=193). Metabolites included are the same as in Figure 4b of main manuscript. Species are annotated on the side with variable(s) they were associated with in ANCOM2 in the respective subgroup (non-diabetics or diabetics), and direction of association with kidney health. Metabolites are annotated on the top with their super-pathway classification, and direction of correlation with eGFR in the respective subgroup (only if q<0.05 for correlation with eGFR). *q<0.05.


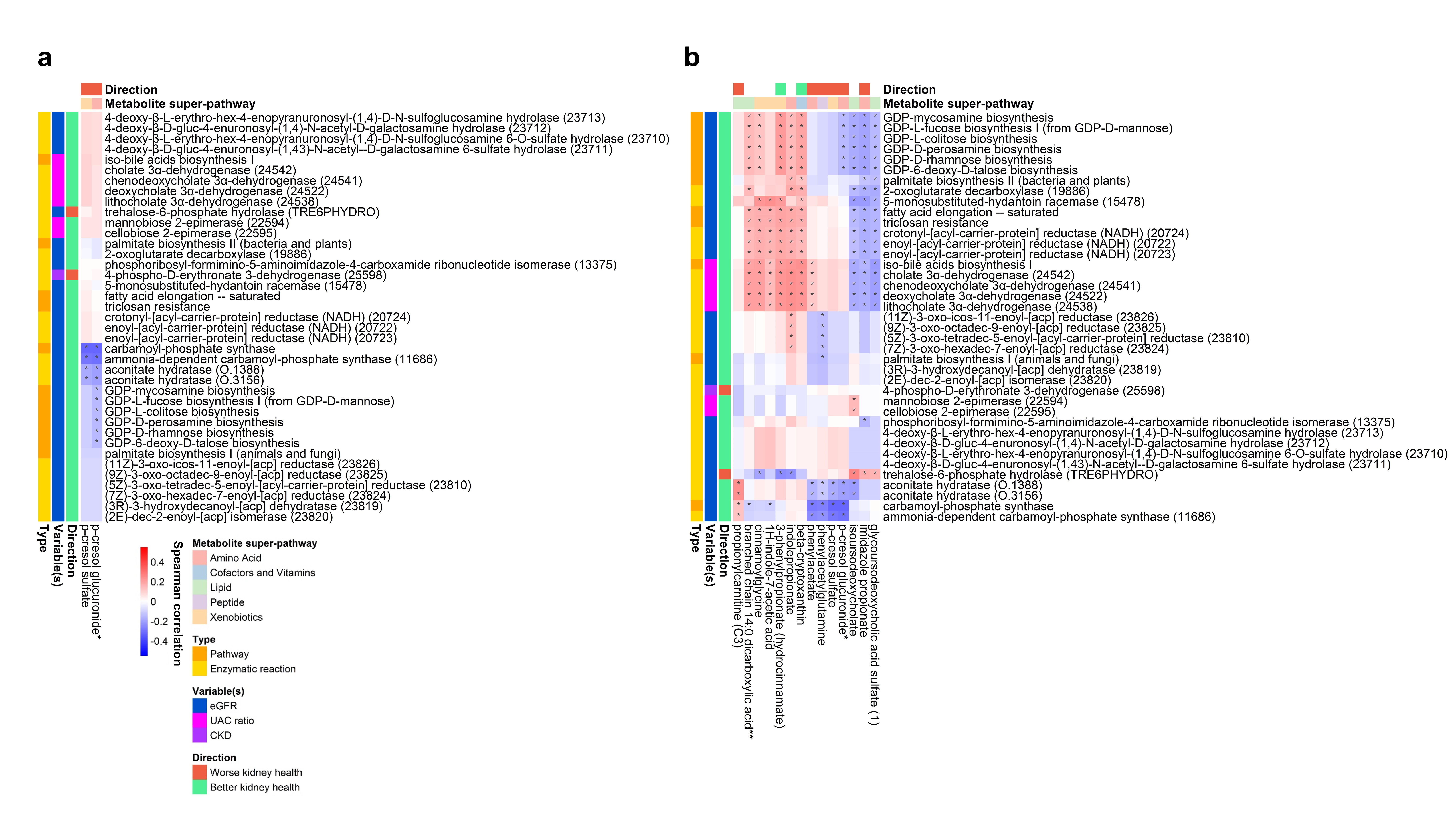


**Supplementary Figure 8.** Spearman correlations of clr-transformed functional pathways and enzymatic reaction abundance with inverse-normal transformed serum metabolites (n=700). Only metabolites that were correlated with at least 1 of these pathways/reactions (q<0.05) with (a) |r|≥0.3 or (b) |r|≥0.2 were included in the heatmaps. Pathways/reactions are annotated on the side with type (pathway or reaction), variable(s) they were associated with in ANCOM2, and direction of association with kidney health. Metabolites are annotated on the top with their super-pathway classification, and direction of correlation with eGFR (only if q<0.05 for correlation with eGFR). *q<0.05.


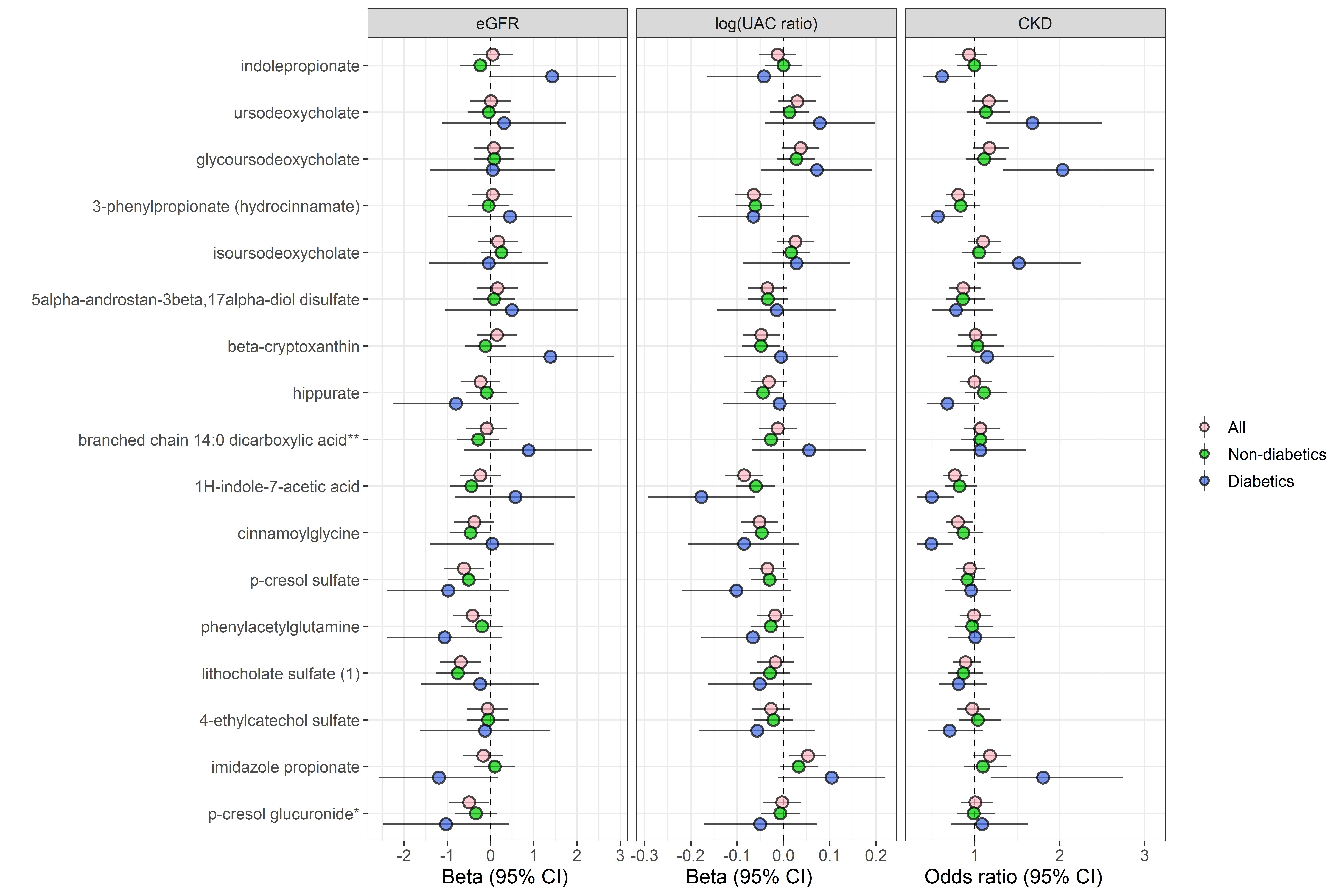


**Supplementary Figure 9.** Prospective association of kidney-microbiome-related metabolites with eGFR, UAC ratio, and incident CKD, restricting to subset randomly selected for metabolomics analysis. For the continuous outcomes of eGFR and UAC ratio, multivariable linear mixed-effects regression models were used to estimate the effect of inverse-normal transformed metabolites at HCHS/SOL visit 1 on eGFR and UAC ratio progression from HCHS/SOL visit 1 to visit 2, adjusting for the following visit 1 covariates: age, sex, field center, Hispanic/Latino background, U.S. nativity, income, educational attainment, cigarette smoking, alcohol use, AHEI2010, predicted sodium intake, predicted protein intake, protein supplement use, total physical activity, BMI, waist-to-hip ratio, systolic blood pressure, diastolic blood pressure, triglycerides, HDL cholesterol, fasting glucose, hypertension medication, diabetes medication, and lipid-lowering medication. Betas are from the interaction of time x metabolite. For the binary outcome of incident CKD, multivariable logistic regression was used to estimate the effect of inverse-normal transformed metabolites at HCHS/SOL visit 1 on incidence of CKD at visit 2, adjusting for the visit 1 covariates listed above. Analyses were performed in all available participants (n=2,116), non-diabetics (n=1,776), and diabetics (n=340).
